# Discovery of two highly divergent negative-sense RNA viruses associated with the parasitic nematode, *Capillaria hepatica*, in wild *Mus musculus* from New York City

**DOI:** 10.1099/jgv.0.001315

**Published:** 2019-09-12

**Authors:** Simon H. Williams, Xiaoyu Che, Alexandra Oleynik, Joel A. Garcia, Dorothy Muller, Tanja S. Zabka, Cadhla Firth, Robert M. Corrigan, Thomas Briese, Komal Jain, W. Ian Lipkin

**Affiliations:** ^1^​ Center for Infection and Immunity, Columbia University, New York, NY, USA; ^2^​ Development Sciences Safety Assessment, Genentech, Inc., South San Francisco, California; ^3^​ Australian Institute of Tropical Health and Medicine, James Cook University, Cairns, Australia; ^4^​ RMC Pest Management Consulting, Briarcliff Manor, New York, USA

**Keywords:** *Capillaria hepatica*, nematode, virome, *Mus musculus*, New York City, wild house mice, Amsterdam virus, Fulton virus, bunyavirus, mononegavirus

## Abstract

Recent advances in high-throughput sequencing technology have led to a rapid expansion in the number of viral sequences associated with samples from vertebrates, invertebrates and environmental samples. Accurate host identification can be difficult in assays of complex samples that contain more than one potential host. Using unbiased metagenomic sequencing, we investigated wild house mice (*Mus musculus*) and brown rats (*Rattus norvegicus*) from New York City to determine the aetiology of liver disease. Light microscopy was used to characterize liver disease, and fluorescent microscopy with *in situ* hybridization was employed to identify viral cell tropism. Sequences representing two novel negative-sense RNA viruses were identified in homogenates of wild house mouse liver tissue: Amsterdam virus and Fulton virus. *In situ* hybridization localized viral RNA to *Capillaria hepatica,* a parasitic nematode that had infected the mouse liver. RNA from either virus was found within nematode adults and unembryonated eggs. Expanded PCR screening identified brown rats as a second rodent host for *C. hepatica* as well as both nematode-associated viruses. Our findings indicate that the current diversity of nematode-associated viruses may be underappreciated and that anatomical imaging offers an alternative to computational host assignment approaches.

## Introduction

Advances in sequencing technology have accelerated the discovery of sequences representing potential new viruses [1]. Recent studies highlight the potential for the discovery and characterization of viruses sourced from diverse origins, including arthropods [2], mammals [3] and marine samples [4]. Viruses have also been detected using high-throughput sequencing (HTS) in investigations of plant-parasitic [5], free-living [6] and vertebrate-parasitic nematodes [7]. Collectively, microbial discovery and surveillance studies provide valuable information pertaining to virus ecology, diversity and evolution. However, it remains difficult to associate a host with a viral sequence from complex samples wherein a variety of organisms may be present [8].

We describe here an investigation of liver disease in wild house mice (*Mus musculus*) trapped in New York City (NYC). Unbiased HTS analysis of extracts of mouse liver led to the discovery of two novel viruses: Amsterdam virus (AMSV) and Fulton virus (FULV). *In situ* hybridization (ISH) demonstrated that nucleic acids of both viruses were present in *Capillaria hepatica* nematodes infecting mouse livers rather than mouse liver parenchyma. Nucleic acids of AMSV and FULV were also detected in nematodes by PCR in the livers of brown rats (*Rattus norvegicus*) from nearby housing complexes in NYC.

## Methods

### Fieldwork and processing

**Fig. 1.**
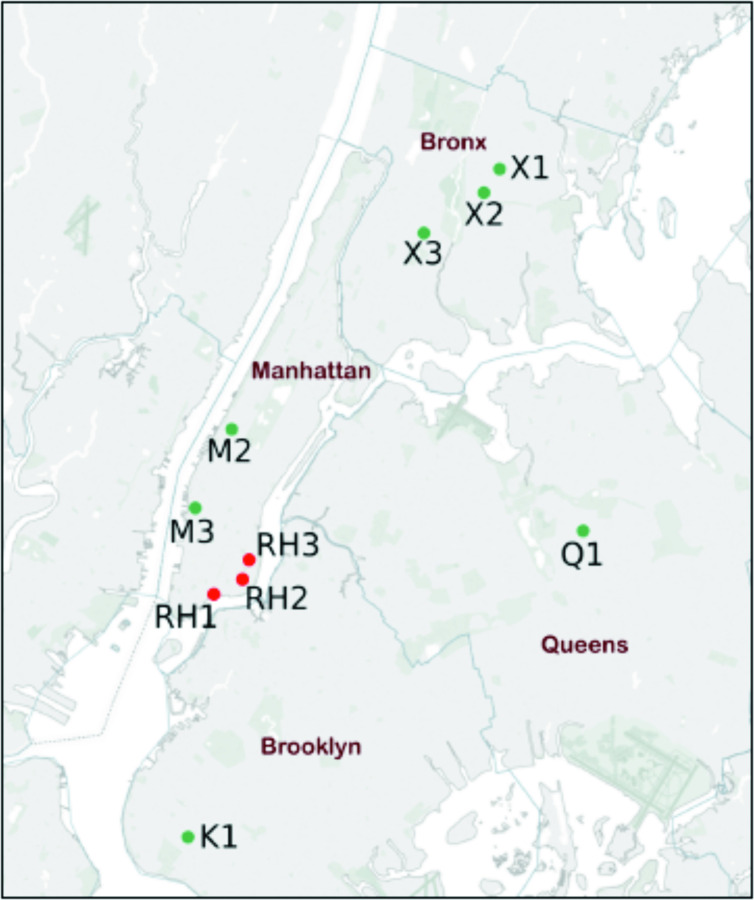
Map of New York City with house mouse (green dot) and brown rat (red dot) trap locations.

Details of the collection and processing of the mice used in this study have been reported previously [9, 10]. House mice were trapped between August 2014 and September 2015. All procedures involving the use of mice were performed with the approval of the Institutional Animal Care and Use Committee at Columbia University (protocol number AC-AAAE8351/AC-AAAE8450). Liver RNA and DNA were purified using the AllPrep DNA/RNA Minikit (Qiagen, Valencia, CA, USA). For the purposes of expanded PCR screening analysis, we also assessed samples from 55 wild brown rats (*Rattus norwegicus*) that were captured from 3 housing sites between September 2012 and June 2013, as described by Firth *et al*. [3]. The trapping locations for house mice were located in Manhattan (M2 and M3), the Bronx (X1, X2 and X3), Queens (Q1) and Brooklyn (K1); brown rats were collected within Manhattan (RH1, RH2 and RH3) (Fig. 1).

### High-throughput sequencing

The RNA from liver samples of five mice, two of which had liver enlargement and multifocal to coalescing pallor, was treated with rDNase 1 (Ambion, Foster City, CA, USA) and Ribo-Zero (Illumina, San Diego, CA, USA) prior to reverse transcription using SuperScript III (Invitrogen, Carlsbad, CA, USA) with random hexamer primers. The cDNA was RNase H-treated prior to second-strand synthesis using Klenow Fragment (3′−5′ exo-) (New England Biolabs, Beverly, MA, USA). Double-stranded cDNA was sheared to an average length of 200 nucleotides (nt) using the Focused-Ultrasonicator E210 (Covaris, Woburn, MA, USA), purified and prepared for paired-end sequencing on the HiSeq 2500 system (Illumina) using the Hyper Prep kit (Kapa Biosystems, Boston, MA, USA). In an attempt to complete partial viral genomes that were identified in this first HTS run, we prepared a second library consisting of two samples that were found to have the lowest cycle threshold value (highest relative viral titre) based upon SYBR Green quantitative PCR (Applied Biosystems, Foster City, CA, USA) for AMSV (Table 1). The second sequencing run was performed using the Ion Personal Genome Machine (PGM) system (Life Technologies, South San Francisco, CA, USA). Double-stranded cDNA was prepared from liver RNA using the same strategy described above for Illumina sequencing, and fragmented using the Ion Shear Plus Reagents kit. Ion Xpress Adapters and unique Ion Xpress Barcodes were ligated to sheared double-stranded cDNA and then amplified with the Ion Plus fragment library kit. Barcoded libraries were bound to Ion Sphere particles using the Ion OneTouch 200 Template kit v2. Emulsion PCR was performed on the Ion OneTouch 2 instrument and template-positive particles were subsequently isolated on the Ion OneTouch ES instrument. Sequencing was performed using the Ion PGM 200 kit v2.

**Table 1.** PCR primers and cycling conditions

Agent	Target gene	Forward primer (5′−3′)	Reverse primer (5′−3′)	Product size (nt)	AT (°C)
FULV FULV FULV	L segment M segment S segment	CTTTCTCACCTGTTGCTACC AGGACTCTGGCAACAAGGC CATACCGTCGATTCATGGCC	GGTCTAATGCAGTATCTGGC ACGACAGTATTGCGCACCAC TTACCATCGGACATCTGCCG	312 239 277	60–55 61–56 61–56
AMSV AMSV qPCR	L protein L protein	GAACCTTCTGCTGGAAGGG TTTTCCTCCGGACAATCGGC	ATCGTCCCATGAAGTCGG GTATGAGCGTAGAAGCGACC	379 157	61–56 58
*C. hepatica*	18S	CCCGTCCCGAACACTGTCA	CTGACCTCTTCAAGACAGGC	309	59

AT, PCR annealing temperature; range of temperatures (xx–yy) indicates touchdown PCR (delta 0.5°C/cycle for 10 cycles).

Sequence reads were trimmed and assessed for quality using PRINSEQ v0.20.2 [11] prior to host genome (mouse mm10 reference genome [12]) subtraction (Bowtie 2 [13]) and assembly (MIRA, v4.0 [14]). The resulting contiguous sequences (contigs) and unique singletons were subjected to similarity search using Megablast and blastx against the non-redundant protein or nucleotide sequence databases of GenBank. All sequences sharing similarity with members of the orders *Mononegavirales* or *Bunyavirales* (maximum E-value of 5.0×10^−3^) were manually edited to remove misassembled termini and *de novo* reassembly was performed using Geneious v7.1.5 [15]. Gaps between contigs were filled using PCR. Final genomes were re-sequenced using overlapping PCR and confirmed by bidirectional Sanger sequencing. Genome termini were generated using rapid amplification of cDNA ends (RACE) with the SMARTer RACE cDNA amplification kit (Clontech, Mountain View, CA, USA).

To investigate the depth of sequence data obtained for FULV and AMSV, we mapped reads from both HTS runs against the genomic sequences of FULV and AMSV with Bowtie 2 [13]. The bam files were parsed using BEDTools (v2.26.0).

### PCR screening

PCR assays for FULV and AMSV, both targeting the RNA-dependent RNA polymerase (RdRp), were performed on liver cDNA for all house mice and brown rats. For FULV, two additional screening assays that target the M and S segments were employed on house mouse samples when the L segment was detected at a particular trapping location. We screened liver DNA for *C. hepatica* using a PCR assay designed to target a conserved region within the 18S rRNA gene. The PCR primers and cycling conditions are described in Table 1. The identity of all PCR products was confirmed by Sanger sequencing.

We also assessed the impact of *C. hepatica* infection on the house mouse virome. Viral screening data were obtained from a prior study where house mouse livers and anal swabs were tested by PCR against 15 viruses identified by HTS from faecal material [9]. Raw sequence data for the 2018 study are also available under BioProject number PRJNA555205. The panel of 15 viruses included a polyomavirus (Mus musculus polyomavirus 3, GenBank accession number MF175082), arterivirus (lactate dehydrogenase-elevating virus, MF416405), coronavirus (murine hepatitis virus, MF416379), reovirus (murine rotavirus, MF416392–MF416402), rhabdovirus (murine faeces-associated rhabdovirus, MF175079), hepe-like virus (murine faeces-associated hepe-like virus, MF175081), 2 astroviruses (murine astrovirus 1, MF175075; murine astrovirus 2, MF175073), 2 caliciviruses (murine sapovirus, MF175077; murine norovirus, MF416380), 2 picornaviruses (murine picornavirus, MF175072; murine kobuvirus 1, MF175074) and 3 parvoviruses (murine-associated porcine bocavirus, MF175076; murine chapparvovirus, MF175078; murine bocavirus; MF175080).

### Light and fluorescent microscopy with ISH

Formalin-fixed mouse liver tissue from five mice (three diseased and two non-diseased) was routinely processed using haematoxylin and eosin-stained sections for examination by light microscopy. Sections from three mice (two diseased and one non-diseased liver) were also processed for fluorescent microscopy using DAPI to highlight cell nuclei and ISH to assess cell tropism for FULV and AMSV using the ViewRNA ISH Tissue 1-Plex assay (Affymetrix, Santa Clara, CA, USA). The branched DNA method employed in the ViewRNA ISH kit is similar to the RNAScope kit (Advanced Cell Diagnostics, Hayward, CA, USA) and is capable of detecting low copy number targets within formalin-fixed paraffin-embedded samples [16]. We employed a 10 min heat pretreatment and 20 min protease digestion and otherwise followed assay guidelines. Slides were mounted with VECTASHIELD HardSet Mounting Medium with DAPI (Vector Laboratories, Burlingame, CA, USA). ISH probes were designed in the RdRp region from each viral genome. Mouse beta-actin and polyomavirus (NJPyV-2013) probes were included as controls. To estimate the percentage of infected nematode eggs, we counted the number of eggs demonstrating characteristic fluorescence using either FULV or AMSV probes. Estimates were generated using a single section of a highly infected liver.

### Viral genomic characterization

Potential signalase cleavage sites were predicted using SignalP 5.0 [17]. Hydrophobic domain predictions were performed using TMHMM 2.0 (http://www.cbs.dtu.dk/services/TMHMM-2.0).

### Phylogenetics

Reference viral sequences were selected using the 2018 version of the International Committee on Taxonomy of Viruses database (http://talk.ictvonline.org), and sourced from the National Center for Biotechnology Information (NCBI) reference sequence database. We also included recently submitted sequences from the most closely related viruses using blast similarity searches. The RdRp protein domain sequences for mononegaviruses and bunyaviruses (pfam00946 and pfam04196, respectively) were aligned using muscle in Geneious 10.2.3 [15], and exported to mega6 [18], where model selection was performed. Maximum-likelihood trees were constructed using the Le and Gascuel substitution model [19] with 500 bootstrap repetitions. Newick trees were exported to FigTree (http://tree.bio.ed.ac.uk/software/figtree/) for annotation.

### Statistical analyses

Statistical data analyses were conducted using Matlab and Statistics Toolbox release 2013a (The MathWorks, Natick, MA, USA). We controlled the family-wise error rate at the 0.05 level using Hochberg’s step-up procedure [20]. All *P*-values are two-tailed.

Associations between *C. hepatica* infection and demographic variables were tested in logistic regression models. With sparse data due to the rareness of nematode infection, we used a penalized likelihood-based method (Firth logistic regression) [21] for unbiased estimation. We also used Firth logistic regression models to test the association between the nematode and nematode-associated viruses, and between nematode and other viral sequences identified from a prior study using the same mouse population [9]. The demographic variables of sex, body weight, body length (a surrogate measure for age) and trapping site were adjusted for as confounding variables in the logistic regression models.

### Accession numbers

Sequences for each virus are available using the GenBank accession numbers MK618652–54 (FULV) and MK618655 (AMSV). Illumina and Ion Torrent PGM sequence data are available under BioProject number PRJNA555205.

## Results

### Virus identification

Sequencing of a pool of five house mouse liver samples using the HiSeq 2500 system (Illumina) yielded a total of 127.5 million reads, of which 14.9 million reads were available for downstream analysis after quality filtering and host subtraction. All trimmed reads were 100 nt long. The assembly of reads generated 139 904 contigs (average length: 325 nt) and 8.2 million unassembled unique singletons. A total of 7190 sequences (0.09 %) (contigs and unique singletons) shared identity with viral sequences using blast similarity comparisons. Data from the second HTS run using the PGM system generated a total of 4.41 million reads with an average length of 140 nt (range 25 to 363 nt), and 1.21 million reads remained after quality filtering and host subtraction. We assembled 24 072 contigs (average length: 331 nt) and identified 822 699 unique singletons from these data; 866 (0.10 %) sequences shared identity with viral sequences.

We mapped reads to the PCR-confirmed genomes of AMSV and FULV to assess the depth of sequence data obtained for each sequencing approach. Mapping of reads generated by Illumina sequencing covered 99.62 % of the final PCR-confirmed AMSV genome (average depth=6.3), while sequencing on the PGM provided 99.90 % (average depth=19.7). Both sequencing methods also obtained near 100 % of the three genome segments for FULV (99.6 to 100 %). The average depth obtained from PGM sequencing for FULV was consistently higher across each segment (L: PGM 66.7, Illumina 46.3; M: PGM 30.9, Illumina 30.5; S: PGM 26.1, Illumina 18.3).

### Virus characterization

Pooled HTS data from HiSeq and PGM runs identified three viruses: lactate dehydrogenase-elevating virus (LaDV) and two highly divergent negative-sense viruses that are apparent members of the orders *Mononegavirales* and *Bunyavirales*. The latter two were tentatively named Amsterdam virus (AMSV) and Fulton virus (FULV) after the neighbourhoods in NYC near which the mice were trapped, leading to their initial identification. LaDV, a common infection of house mice, was 88 % similar at the nt level to the LaDV Plagemann strain across the available 14 052 nt sequence. The distribution and prevalence of LaDV among wild mice in NYC has been described by Williams *et al*. [9].

#### Fulton virus

We obtained three genomic segments for FULV representing the L, M and putative S sequences that shared low amino acid (aa) sequence identity with members of the order *Bunyavirales*. The RdRp encoded in the L segment shared 25 % aa identity to Nyando virus, an orthobunyavirus carried by *Anopheles* mosquitoes [22]. At 2574 aa, the L protein is longer than known for peribunyaviruses (Table 2). The L protein was characterized by conservation of three domains: bunyavirus RdRp (pfam04196, aa positions 675–1578, E-value 1.54×10^−62^), L protein N-terminus (pfam15518, 55–134, E-value 4.89×10^−4^) and DNA polymerase III PolC (prk00448, 1009–1243, E-value 8.99×10^−3^).

**Table 2.** Protein lengths for Fulton virus and members of the family *Bunyavirales*

		L segment		M segment			S segment		
Viral family	Genus/virus	Accession	RdRP (aa)	Accession	Glycoprotein precursor (aa)	NSm (aa)	Accession	Nucleocapsid (aa)	NSs (aa)
	Fulton virus		2574		1486*$	(130)		253	$
*Peribunyaviridae*	*Orthobunyavirus*								
	Bunyamwera virus^#^	NC001925	2238	NC001926	1433*	(167)	NC001927	233	101
	Nyando virus	NC034492	2268	NC034491	1432*	(165)	NC034481	233	92
	Anopheles A virus	KY793537	2242	KY793538	1405*	(158)	KY793539	245	–
	*Herbevirus*								
	Herbert virus^#^	NC038714	2423	NC038712	831	–	NC038712	226	–
	Kibale virus	NC034460	2422	NC034458	830	–	NC034458	226	–
	Tai virus	NC034459	2425	NC034457	838	–	NC034457	225	–
	*Shangavirus*								
	Shuangao insect virus 1	NC031221	2335	NC031223	1539*	(259)	NC031223	270	–
*Cruliviridae*	*Lincruvirus*								
	Wenling crustacean virus 9^#^	NC032143	2173	NC032145	914	–	NC032145	212	–
*Tospoviridae*	*Orthotospovirus*								
	Tomato spotted wilt virus^#^	NC002052	2875	NC002051	1135	303	NC002051	258	464
	Bean necrotic mosaic virus	NC018070	2932	NC018071	1161	317	NC018071	270	439
	Iris yellow spot virus	NC029799	2873	NC029800	1136	311	NC029800	273	443
*Fimoviridae*	*Emaravirus*								
	European mountain ash ringspot-associated emaravirus^#^	NC013105	2293	NC013108	646	–	NC013108	314	–
							
	Rose rosette virus	NC015298	2276	NC015300	645	–	NC015300	316	–
	High plains wheat mosaic virus	NC029570	2272	NC029550	647	–	NC029550	289	–
*Hantaviridae*	*Orthohantavirus*								
	Hantaan orthohantavirus^#^	NC005222	2151	NC005218	1135	–	NC005218	429	–
*Phasmaviridae*	*Feravirus*								
	Ferak virus*	KP710246	2271	KP710267	1262	109^	KP710267	306	107

#, type species; *, NSm encoded within glycoprotein precursor; $, incomplete sequence; ^, putative NSm encoded in −1 frameshift relative to glycoprotein precursor with 32 nt overlap.

**Fig. 2.**
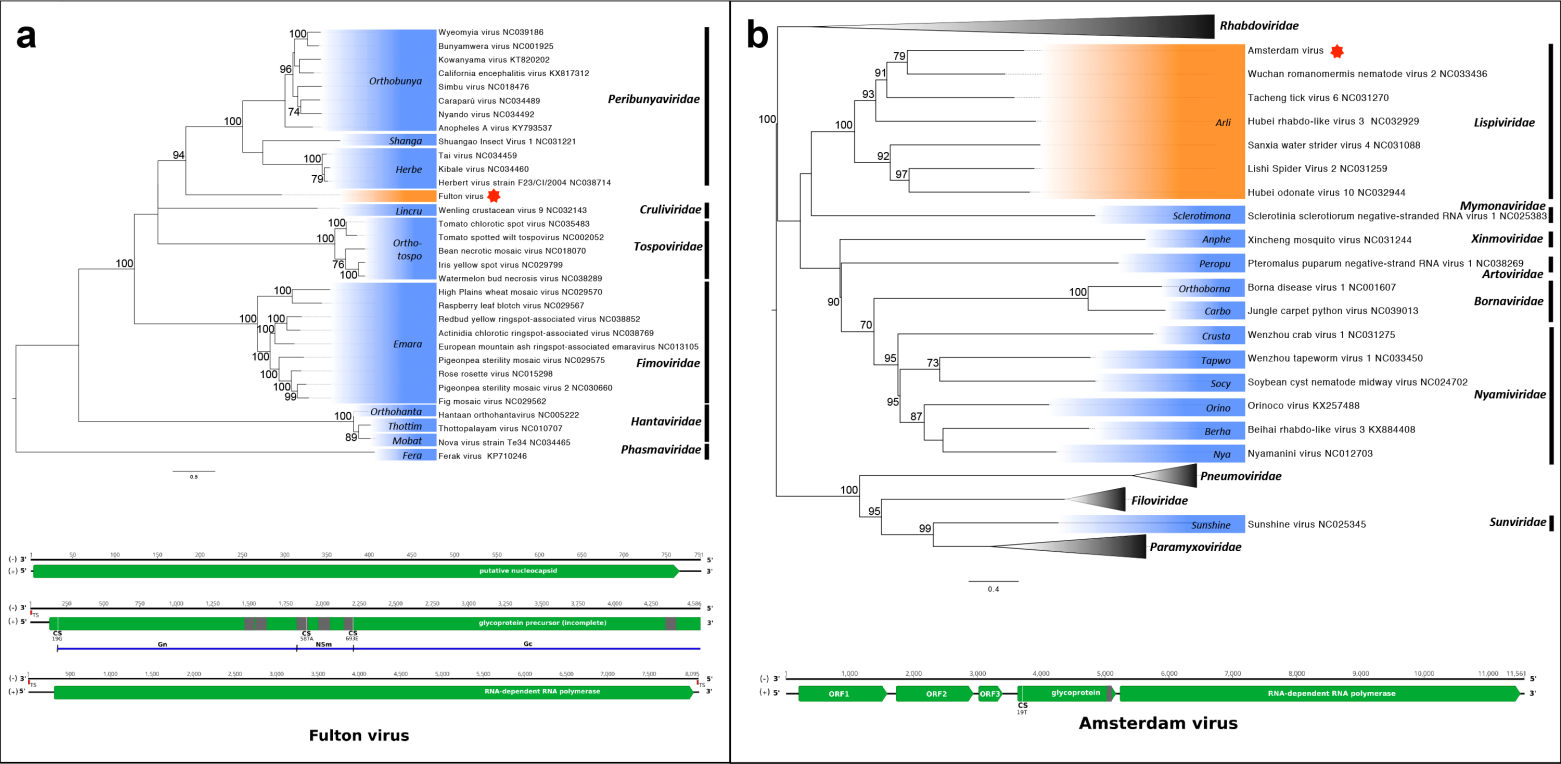
Phylogenetic analysis and genome organizations of Fulton virus (a) and Amsterdam virus (b). Maximum-likelihood trees were constructed using RdRp protein domain sequences for mononegaviruses and bunyaviruses (pfam00946 and pfam04196, respectively). The scale bar represents substitutions per site and bootstrap support values are displayed when greater than 70 %. Representative viral genera are highlighted in blue. Viruses identified in this study are marked by a red star and the proposed genera are highlighted in orange. Genome illustrations indicate putative open reading frames (green arrows). Genome length is not to scale; numbers indicate nucleotide position. Terminal sequences (TSs) for Fulton virus are indicated with red boxes. Glycoproteins are labelled with hydrophobic domains (grey shading) and predicted cleavage sites (CS) with corresponding amino acid position. The Fulton virus N-terminal glycoprotein (Gn), non-structural movement protein (NSm) and C-terminal glycoprotein (Gc) are demarcated in blue.

The glycoprotein precursor (GPC) amino acid sequence encoded by the M segment was 22 % identical at the aa level to the GPC protein of impatiens necrotic spot virus (INSV), a plant tospovirus. The GPC was characterized by three conserved domains: bunyavirus glycoprotein G1 (pfam03557, 1018–1307, E-value 8.81×10^−19^), DNA anti-recombination protein RmuC (COG1322, 769–905, E-value 2.60×10^−3^) and chromosome segregation protein SMC (TIGR02169, 757–910, E-value 3.05×10^−3^). A signal peptide of 19 aa is predicted to be cleaved after G_19_ at the N-terminus of the precursor. The FULV M segment contained a single open reading frame (ORF) (>4458 nt in length), whereas the M segment from INSV encodes a 3333 nt ORF (glycoprotein) and a non-overlapping 912 nt ambisense ORF (non-structural movement protein NSm), typical of the genus *Orthotospovirus*, family *Tospoviridae*. As we could not complete the sequence at the 5′ terminus of the genomic sense FULV M segment, we cannot exclude the possibility that an ambisense ORF exists beyond the extent of our sequence. However, the predicted length of the FULV GPC protein (1486 aa; Table 2) and alignments with orthobunyavirus GPCs suggested that an NSm protein is encoded between a Gn and Gc polyprotein. A nested position of NSm relative to Gn and Gc, and the cleavage of the GPC by cellular signalases, is characteristic of orthobunyaviruses [23]. We identified putative signalase cleavage motifs after A_587_ and A_693_, with each cleavage site predicted to occur at the C-terminal end of hydrophobic transmembrane domains (Fig. 2a). A total of three predicted hydrophobic domains were contained within the putative NSm, similar in location and length to the transmembrane domains found in other orthobunyaviruses (Fig. 2a). We also analysed the M segment sequence of the recently described Shuangao insect virus 1 (SgIV-1). SgIV-1 is the type species of the genus *Shangavirus*, family *Peribunyaviridae*, and was detected in an insect mix sourced from green lacewings (*Chrysopidae*) and drain flies (*Psychoda alternata*) [2]. SgIV-1 appears to encode an elongated putative NSm protein nested between Gn and Gc (Table 2) with putative cleavage sites identified after G_319_ and A_557_, and three characteristic hydrophobic domains (data not shown). At the time of writing, the genus *Herbevirus* is a third genus of family *Peribunyaviridae*, while a fourth genus, *Pacuvirus,* has just recently been included [24]. Unlike peribunyaviruses and presumably shangaviruses, herbeviruses lack an NSm protein. Taken together, these data suggest that the FULV GPC shares structural similarities with orthobunyaviruses and SgIV-1.

We were unable to identify protein sequences with similarity to bunyavirus nucleocapsid proteins encoded in the S segment using default blastp similarity searches within GenBank. However, using the uniprotkb database (www.uniprot.org/blast), we identified a 136 aa peptide that shared 30 % identity with the capsid protein from High Plains virus Kansas/04 (now named High Plains wheat mosaic virus), a member of the genus *Emaravirus* (family *Fimoviridae*). Using PCR to fill gaps between candidate sequences, we obtained a 791 nt genomic S segment. There was a single, complete 762 nt ORF encoding a 253 aa protein in the S segment, but as we were not able to sequence either of the segment termini, we cannot exclude the possibility that an ambisense ORF exists elsewhere in the S segment (Table 2). Using PCR, we confirmed the presence of this putative S segment sequence in 90 % (9/10) of L segment-positive and M segment-positive samples and we did not detect this sequence in any L segment-negative and M segment-negative samples.

Despite repeated attempts to generate a complete genomic sequence using RACE, we were unable to obtain the 5′ terminus of the negative M segment strand, or either terminus for the S segment. Bunyaviruses have characteristic conserved terminal sequences that are unique to members of the same genus, but different from those of other genera. The sequence UCAUCAAUUG was identified at the 3′ terminus and AGUAGUAUUC was identified at the 5′ terminus of the negative-sense L segment; the sequence for the 3′ terminus of the negative-sense M segment was UCAUCAGUUG. These terminal sequences share greatest identity with emaraviruses (Table 3).

**Table 3.** Terminal sequences of Fulton virus and other selected members of family *Bunyavirales*

Viral family	Virus	Sequence 3′	Sequence 5′
	Fulton virus L segment	UCAUCAAUUG	AGUAGUAUUC
	Fulton virus M segment	......G...	na
*Peribunyaviridae*	*Orthobunyavirus*		
	Bunyamwera virus^#^	UCAUCACAUG	AGUAGUGUGC
	Nyando virus	..........	..........
	Anopheles A virus	..........	..........
	*Herbevirus*		
	Herbert virus^#^	UCAUCACACG	AGUAGUGUGC
	Kibale virus	.......GU.	.......C..
	Tai virus	.......GU.	..........
*Cruliviridae*	*Lincruvirus*		
	Wenling crustacean virus 9^#^	UCAUCAUAUG	AGUAGUAGAC
*Tospoviridae*	*Orthotospovirus*		
	Tomato spotted wilt virus^#^	UCUCGUUAGU	AGAGCAAUCA
	Bean necrotic mosaic virus	.........C	.........G
	Iris yellow spot virus	.......CAG	.........G
*Fimoviridae*	*Emaravirus*		
	European mountain ash ringspot-associated emaravirus^#^	UCAUCACUUG	AGUAGUGUUC
	Rose rosette virus	.......AA.	.......AA.
	High plains wheat mosaic virus	.......A..	.......A..
*Hantaviridae*	*Orthohantavirus*		
	Hantaan orthohantavirus^#^	AUCAUCAUCU	UAGUAGUAGU
*Phasmaviridae*	*Feravirus*		
	Ferak virus^#^	UCAUCAUUUG	AGUAGUAAAC

Termini not available for Shaungao insect virus 1, type species of genus *Shangavirus*, family *Peribunyaviridae*. Reference terminal sequences use L segment where available. #, type species.

Phylogenetic analysis using the conserved bunyavirus RdRp domain (pfam04196) from representative viruses of the most closely related *Bunyavirales* families (*Peribunya-, Cruli-*, *Tospo*-, *Fimo-*, *Hanta-*) places FULV in a monophyletic clade rooted in a posterior position relative to the genera *Orthobunya-, Shanga-* and *Herbevirus* of the family *Peribunyaviridae* (Fig. 2a). Its phylogenetic placement indicates that FULV may represent a novel genus within the order *Bunyavirales*.

#### Amsterdam virus

The genome organization of AMSV contains five ORFs, including the L protein that encodes the viral RdRp. AMSV shares 35 % aa identity with the L protein of Wuchan romanomermsis nematode virus 2, but lacks similarity across the remaining proteins when using blast similarity searches. We identified four conserved domains within the predicted 2089 aa L protein, including mononegavirales RdRp (pfam00946, aa positions 24–1034; E-value 3.01×10^−140^), mRNA (guanine-N7-)-methyltransferase (TIGR04198, 1142–1884, E-value 1.13×10^−28^), mononegavirales mRNA capping region V (pfam14318, 1048–1279, E-value 3.40×10^−28^) and 23S rRNA U2552 (ribose-2′-O)-methylase RimE/FtsJ (COG0293, 1764–1871, E-value 6.85×10^−6^). We also identified a putative 512 aa glycoprotein (ORF4) with a class I transmembrane domain located between aa positions 467 and 489. A signal peptide of 19 aa is predicted to be cleaved after T_19_ at the N-terminus (Fig. 2b).

Phylogenetic analysis indicated that AMSV clustered most closely with a virus sourced from a nematode, Wuchan romanomermis nematode virus 2 (Fig. 2b). AMSV groups within the newly established genus *Arlivirus* of the family *Lispiviridae* (order *Mononegavirales*) [25], along with other viruses identified in a metagenomic study of arthropods in China [2].

#### Viral and nematode prevalence

The occurrence of the nematode (*C. hepatica*) in trapped house mice was limited to three sites: M2, M3 and K1, with prevalences of 12, 16 and 80 %, respectively (Table 4). The nematode was not detected in any of the sites in Queens or the Bronx, despite these sites providing over half of the mice investigated in this study. Adult mice accounted for 79 % of all *C. hepatica* infections. A higher mouse body weight was associated with an increased likelihood of *C. hepatica* detection [odds ratio=1.254; 95 % confidence interval (CI), 1.057 to 1.498; *P* *=*0.009], regardless of the mouse length, which was used to infer age. The prevalence of *C. hepatica* may be underestimated in our study, as infection may sometimes be localized in regions of the liver that were not tested by PCR.

**Table 4.** Nematode and viral prevalence in house mice

Borough	Site	*n*	*C. hepatica*	AMSV	FULV L segment	FULV M segment	FULV S segment
Manhattan	M2	42	5 (11.9)	4 (9.5)	4 (9.5)	4 (9.5)	4 (9.5)
	M3	119	19 (16.0)	14 (11.8)	6 (5.0)	6 (5.0)	5 (4.2)
Queens	Q1	172	0	0	0	NP	NP
Bronx	X1 X2 X3	38 1 1	0 0 0	0 0 0	0 0 0	NP NP NP	NP NP NP
Brooklyn	K1	5	4 (80.0)	0	0	NP	NP
Total		378	28 (7.4)	18 (4.8)	10 (2.7)	10	9

NP, not performed.

FULV and AMSV sequences were detected only in rodents confirmed to be infected with the nematode. The association between both viruses and *C. hepatica* was statistically supported with an observed odds ratio of 401.8 for AMSV (95 % CI, 49.2 to 51 333.7, *P*=5.11×10^−15^) and 206.9 for FULV (95 % CI, 17.6 to 32 335.7, *P*=1.02×10^−7^) independent of mouse sex, body weight, body length and trapping site. Overall, 64 % of mice infected with *C. hepatica* were AMSV PCR-positive, while 36 % were FULV PCR-positive and 36 % were positive for both viruses. Thus, AMSV was observed independent of FULV, whereas FULV was only observed in association with AMSV. The L, M and S segments of FULV were identified in 9/10 mice. One mouse was only positive for the L and M segments. By aligning sequences obtained from screening PCR products, we observed limited heterogeneity amongst FULV-positive mice (L segment, *p-*distance range=0 to 0.004; M segment, *p-*distance range*=*0 to 0.009; S segment, *p-*distance range*=*0 to 0.002). Sequences from the AMSV PCR targeting the RdRp were 100 % identical; however, PCR targeting the glycoprotein may provide a better assessment of AMSV sequence heterogeneity.

We investigated the prevalence of *C. hepatica* in the brown rat (*Rattus norvegicus*), another ubiquitous potential reservoir. The livers of 54 brown rats from three sites in Manhattan were screened by PCR. Rats from two of these sites were *C. hepatica* DNA-positive, with a prevalence of 58% and 75 % in RH2 and RH3, respectively (Table 5). We found no evidence for *C. hepatica* infection in site RH1. We also detected FULV and AMSV in rat liver, but only in rats that were infected with *C. hepatica*. The prevalence of both FULV and AMSV was lower in rats than in mice. Of 22 *C* *. hepatica*-positive rats, 32 % carried AMSV, 23 % were FULV PCR-positive and 18 % were positive for both viruses. Unlike in mice, we were able to detect FULV in the absence of AMSV in a single rat sample. Sequences from PCR products indicated variation between rats for both FULV (L segment, *p-*distance range=0 to 0.007) and AMSV (*p-*distance range=0 to 0.026); however, both PCR screening assays targeted conserved RdRp sequences and may have underestimate the genetic diversity of each virus in rats.

**Table 5.** Nematode and viral prevalence in brown rats

Borough	Site	*n*	*C. hepatica*	AMSV	FULV L segment
Manhattan	RH1	22	0	0	0
	RH2	12	7 (58.3)	4 (33.3)	3 (25.0)
	RH3	20	15 (75.0)	3 (15.0)	2 (10.0)
Total		54	22 (40.1)	7 (13.0)	5 (9.3)

#### Origins of the viruses

**Fig. 3.**
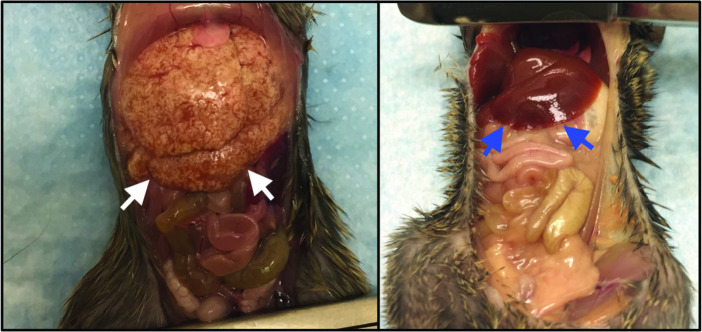
Ventral abdominal cavity view demonstrating an enlarged liver (white arrows) with multifocal to coalescing areas of pallor from a mouse heavily infected with *C. hepatica* (left) compared to a normal liver (blue arrows) from a non-infected mouse (right).

**Fig. 4.**
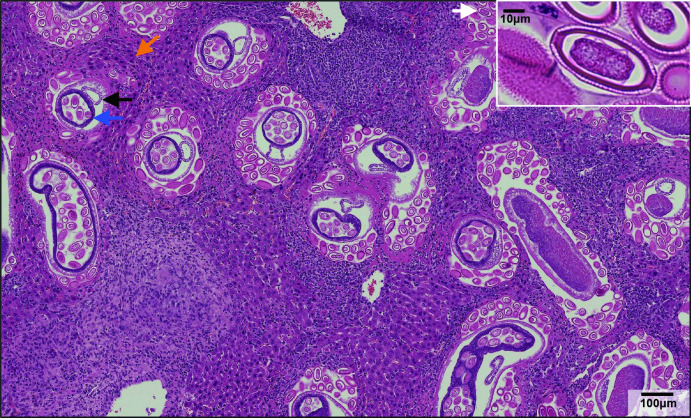
Haematoxylin and eosin-stained section of liver from a mouse heavily infected with *C. hepatica*. Adult worms (blue arrow, ovum containing eggs; black arrow, intestine) and free eggs (white arrow) were located within the liver parenchyma and associated with multifocal to coalescent granulamatomous inflammation with central necrosis and multinucleated giant cells (orange arrow). This microscopic finding corresponded to the enlarged liver and areas of pallor depicted in Fig. 3. Insert: *C. hepatica* egg with characteristic bipolar plugs.

**Fig. 5.**
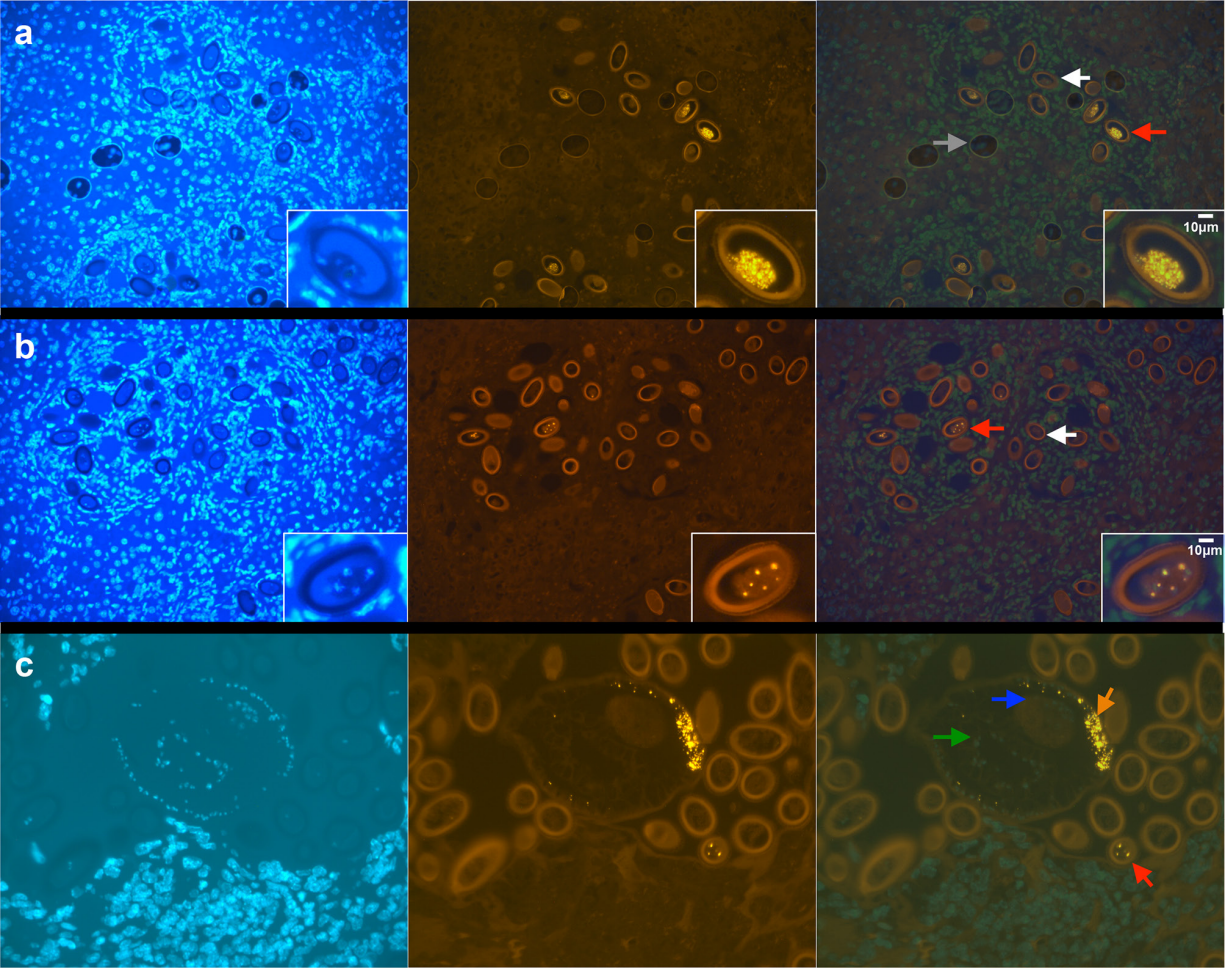
Fluorescent *in situ* hybridization demonstrating the presence of viral RNA within *C. hepatica*: white arrow, uninfected egg; red arrow, viral RNA-positive egg; grey arrow, air bubble; blue arrow, adult ovum; green arrow, adult intestine; orange arrow, AMSV RNA localized in adult nematode; (images from left to right) DAPI stain for cell nucleus, fluorescence ISH for viral RNA and overlay for co-localization. (a) Fulton virus RNA in nematode eggs; insert of higher magnification of virus-infected egg. (b) Amsterdam virus RNA in nematode egg; insert of higher magnification of virus-infected egg. (c) Amsterdam virus RNA in nematode adult and free egg.

During necropsy, we observed house mice with gross liver pathology, including liver enlargement and multifocal to coalescing pallor (Fig. 3). Sections from five mice (three showing disease and two not showing clear signs of disease) were stained using haematoxylin and eosin and examined by light microscopy. The findings noted during necropsy corresponded microscopically to moderate to severe granulomatous hepatitis with multifocal central necrosis and intralesional nematode adults and eggs consistent with *Capillaria* spp. infection (Fig. 4). The eggs were approximately 50–55 µM long and 25–29 µM wide, barrel-shaped, and had bipolar plugs (Fig. 4 insert). FULV and AMSV nucleic acids were observed within *C. hepatica* eggs. FULV RNA was located within eggs, with regionally diffuse distribution in approximately 16 % of eggs (Fig. 5a). In contrast, AMSV RNA had a pinpoint distribution and was observed in approximately 36 % of eggs (Fig. 5b). AMSV RNA was also observed in the hypodermis tissue of an adult worm (Fig. 5c). The strong localization of viral RNA at the anterior of the adult worm may indicate infection within the bacillary band, a tissue involved in nutrient absorption found in *Trichuris* and *Capillaria* nematodes [26]. However, serial section hemotoxylin and eosin-stained images were not available to confirm this possible localization.

#### Impact of *C. hepatica* on wild *M. musculus* virome

We assessed the impact of *C. hepatica* on the house mouse faecal and liver viromes by combining data obtained from this study with data from a prior study using the same set of mouse samples [9]. *C. hepatica*-positive mice carried more viruses (excluding FULV and AMSV) than *C. hepatica-*negative mice in liver samples (average=1.55 vs 1.13) and anal swabs (2.59 vs 1.98). However, after adjusting for trapping site, body length, body weight and sex, these differences between the viral richness detected in nematode-positive and nematode-negative mice were not statistically significant in either the liver (1.14-fold increase in *C. hepatica*-positive; 95 % CI, 0.832 to 1.562; *P*=0.416) or anal swabs (1.07-fold decrease; 95 % CI, 0.817 to 1.399; *P* *=*0.625).

We failed to identify a statistically significant association between the presence of *C. hepatica* in the faeces or liver with any non-nematode virus (Fig. S1, available in the online version of this article). However, the prevalence for some viruses (lactate dehydrogenase-elevating virus (LaDV), murine chapparvovirus, murine-associated porcine bocavirus, murine astrovirus 2 and Mus musculus polyomavirus 3) was higher in mice infected with *C. hepatica* in both the liver and faeces relative to mice not infected with *C. hepatica* (Fig. S1). In particular, LaDV prevalence was higher in the liver and faeces of *C. hepatica-*positive mice and showed a substantial effect size (odds ratio=3.84, 95 % CI, 1.21 to 13.67; *P*=0.0216), despite not reaching significance. Conversely, several viruses [murine astrovirus 1, murine sapovirus, murine norovirus, murine kobuvirus, murine picornavirus, murine hepatitis virus (MHV) and murine bocavirus] were more prevalent in the liver and faeces of *C. hepatica-*negative mice. MHV was ninefold more likely to be found in mice that were not infected with *C. hepatica* (odds ratio=0.11, 95 % CI, 0 to 0.86; *P*=0.0323) than in *C. hepatica-*positive mice. The lack of significance observed using these analyses may be due to the rare event of viral infection, but the large effect sizes warrant further investigation.

## Discussion

Sequencing nucleic acid extracts of livers from mice with gross anatomical and histological liver pathology revealed the presence of a novel bunyavirus and a novel lispivirus. ISH demonstrated that viral nucleic acid was localized to nematode adults and eggs within the liver, rather than the liver parenchyma. To the best of our knowledge, viruses have not previously been reported from *C. hepatica*. Viral RNA was present within unembryonated eggs of *C. hepatica*, suggesting that both viruses can be maintained in the nematode population via vertical transmission. We also identified AMSV RNA in an adult worm, providing evidence that AMSV is capable of maintaining infection throughout the multiple stages of the nematode lifecycle. Taken together, the combined HTS and ISH data highlight the importance of characterization beyond HTS to ultimately determine virus host, cell tropism and possible routes of transmission. In the absence of ISH, these novel viruses might have been misassigned as house mouse or brown rat viruses. While not employed in this study, computational methods have been proposed to aid host classification in metagenomic studies. These methods assess several criteria, including the abundance of viral reads as a proportion of total RNA, the presence of related viruses in the same or similar hosts and the co-occurrence of viral reads with non-host reads [27].

*C. hepatica* is a zoonotic parasitic nematode that commonly infects house mice [28], brown rats [29–31] and other incidental hosts, including humans [32]. In mammals, ingestion of embryonated *C. hepatica* eggs precedes larval penetration of the intestine and migration to the liver. Adult worms mate within the hepatic parenchyma, where they deposit eggs that remain in place until the death of the host. Eggs require oxygen to embryonate and are typically infectious after 1 to 2 months, or after passing completely through the gastrointestinal tract of a preying host [32]. The clinical manifestations of *C. hepatica* infection in humans include fever, hepatomegaly and leukocytosis; however, infection is rare, with only 163 cases of hepatic capillariasis reported worldwide through 2011 [33]. Poor hygienic conditions and proximity to rodents have been identified as risk factors [33].

Infection and carriage of viruses by nematodes has been described in plants, insects and fish using classical discovery techniques such as transmission electron microscopy [34–36]. Since the advent of HTS, multiple novel viruses have been described in the nematode model organism, *Caenorhabditis elegans* [6, 37]*,* the soybean cyst nematode (*Heterodera glycines*) [38, 39] and several vertebrate-parasitic nematodes [7]. A virome analysis of pig faeces identified two sequences that shared identity with the *Ascaris suum* transcriptome that, through testing of DNA, were later excluded as from the nematode genome [40]. Instead, these sequences represented posaviruses (order *Picornavirales*) that were most likely infecting their nematode host. ISH has also been used to localize viral nucleic acid within purified nematode cultures [5, 6]. Like the viruses identified in these investigations, the *C. hepatica*-associated viruses described in this study appear to be novel and share little similarity with currently classified viruses. AMSV, whilst clustering within the genus *Arlivirus* of the *Lispiviridae*, shares just 35 % aa identity with its closest relative within the RdRp – the most conserved protein of these viruses. The phylogenetic placement of FULV outside the diverse members of the *Peribunyaviridae* and the unique terminal sequences that share greatest identity with the *Fimoviridae* highlight the novelty of this virus. Taken together, these data indicate that there is a breadth of viral diversity in nematodes that remains to be discovered.

We also assessed the impact of *C. hepatica* infection on viral diversity by combining data from a prior virome analysis with the *C. hepatica* screening data in this study. Nematode-positive mice carried more viruses on average than nematode-negative mice, but these differences were not statistically significant. Helminth infections can induce a type 2 immune response, impairing type 1 responses that target viruses and intracellular bacteria, and type 3 responses that target extracellular bacteria [41]. Controlled experiments wherein mice were infected with helminth parasites led to dampened viral immune control. Higher viral loads of murine norovirus were observed in mice infected with *Trichinella spiralis* [42], and a loss of control of murine gammaherpesvirus-68 was observed in mice challenged with *Heligmosomoides polygyrus* or *Schistosomiasis mansoni* [43]. A study of rats infected with *C. hepatica* did not identify a bias towards the type 1 or type 2 response, suggesting that the immune response may vary according to the host or helminth [44]. The observed increase in viral richness observed in wild mice coinfected with *C. hepatica* warrants further investigation.

## Supplementary Data

Supplementary material 1
