# Supplementary material for: Discovery of two highly divergent negative-sense RNA viruses associated with the parasitic nematode, Capillaria hepatica, in wild Mus musculus from New York City
Source: J Gen Virol. 2019 Sep 12;100(10):1350–62. doi: 10.1099/jgv.0.001315 (PMC7363305; doi:10.1099/jgv.0.001315)
Supplement: Supplementary material 1 [file jgv-100-1350-s001.pdf]

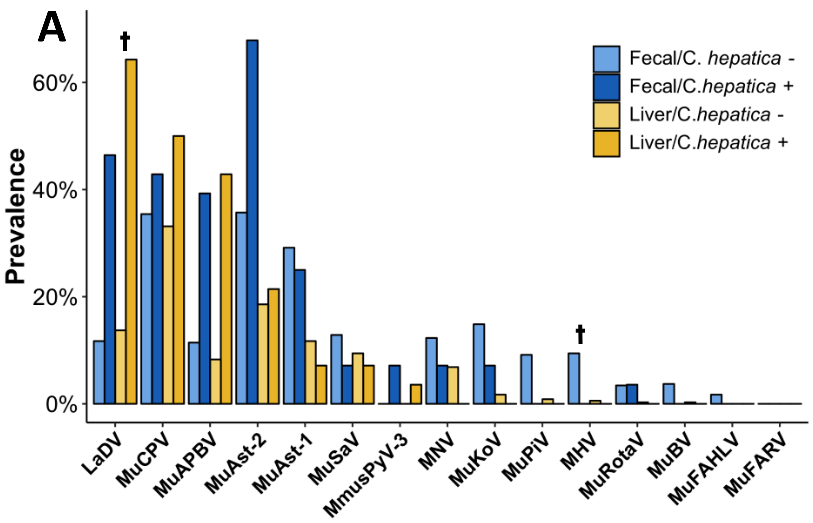


**B**

**Liver**

| Virus | Odds Ratio | 95% CI | P-Value |
|---|---|---|---|
| LaDV | 3.84 | (1.21, 13.67) | 0.0216† |
| MuSaV | 0.77 | (0.14, 2.93) | 0.7208 |
| MuPiV | 0.52 | (0, 16.76) | 0.7467 |
| MuFARV | 2.67 | (0, 1556.18) | 0.7445 |
| MuFAHLV | 2.67 | (0, 1556.18) | 0.7445 |
| MuAst-2 | 0.70 | (0.23, 1.87) | 0.4859 |
| MuAst-1 | 1.33 | (0.25, 5.17) | 0.7062 |
| MHV | 1.53 | (0.01, 38.92) | 0.8309 |
| MuRotaV | 1.33 | (0, 35.01) | 0.8838 |
| MuBV | 7.68 | (0.01, 61504.73) | 0.7364 |
| MuAPBV | 2.81 | (0.93, 9.07) | 0.0682 |
| MuCPV | 0.82 | (0.26, 2.67) | 0.7311 |
| MuKoV | 1.00 | (0.01, 16.71) | 0.998 |
| MmusPyV-3 | 11.02 | (0.04, 6369.63) | 0.3123 |
| MNV | 0.92 | (0, 17.67) | 0.9678 |

**Fecal**

| Virus | Odds Ratio | 95% CI | P-Value |
|---|---|---|---|
| LaDV | 1.57 | (0.53, 4.67) | 0.4109 |
| MuSaV | 0.52 | (0.1, 1.85) | 0.3325 |
| MuPiV | 0.25 | (0, 4.11) | 0.3849 |
| MuFARV | 2.67 | (0, 1556.18) | 0.7445 |
| MuFAHLV | 0.27 | (0, 5.67) | 0.4446 |
| MuAst-2 | 1.51 | (0.55, 4.5) | 0.4328 |
| MuAst-1 | 0.65 | (0.24, 1.64) | 0.3719 |
| MHV | 0.11 | (0, 0.86) | 0.0323 † |
| MuRotaV | 0.67 | (0.06, 3.68) | 0.6686 |
| MuBV | 3.61 | (0.01, 765.94) | 0.5881 |
| MuAPBV | 2.10 | (0.76, 6.01) | 0.1505 |
| MuCPV | 0.88 | (0.33, 2.3) | 0.792 |
| MuKoV | 0.77 | (0.14, 3.01) | 0.725 |
| MmusPyV-3 | 8.79 | (0.03, 2822.61) | 0.426 |
| MNV | 1.02 | (0.13, 6.91) | 0.9847 |

**Supplemental Figure 1**. Association between *Capillaria hepatica* infection and carriage of individual viruses identified by PCR in a prior study of the same mouse population. **(A)** Bar graph displaying prevalence of 15 viruses in the feces or liver of mice with or without *Capillaria hepatica* infection. **(B)** Association between nematode infection and carriage of viruses in the liver or feces. Odds ratios were calculated using Firth logistic regression analysis. †, crude $P < 0.05$, however, after controlling the family-wise error rate, these associations were not significant. LaDV, lactate dehydrogenase-elevating virus; MuSaV, murine sapovirus; MuPiV, murine picornavirus; MuFARV, murine feces-associated rhabdovirus; MuFAHLV, murine feces-associated hepe-like virus; MuAst-2, murine atrovirus 2; MuAst-1, murine astrovirus 1; MHV, murine hepatits virus; MuRotaV, murine rotavirus; MuBV, murine bocavirus; MuAPBV, murine-associated porcine bocavirus; MuCPV, murine chapparvovirus; MuKoV, murine kobuvirus; MmusPyV-3, Mus musculus polyomavirus 3; MNV, murine norovirus
